# Supplementary material for: Temporal Analysis of Embryonic Epidermal Morphogenesis in Caenorhabditis elegans
Source: Int J Mol Sci. 2025 Nov 6;26(21):10802. doi: 10.3390/ijms262110802 (PMC12608371; doi:10.3390/ijms262110802)
Supplement: Supplementary file 1 [file ijms-26-10802-s001.zip › Supplementary Figure.pdf]

# Supplementary Information:

# Temporal Analysis of Embryonic Epidermal Morphogenesis in *Caenorhabditis elegans*

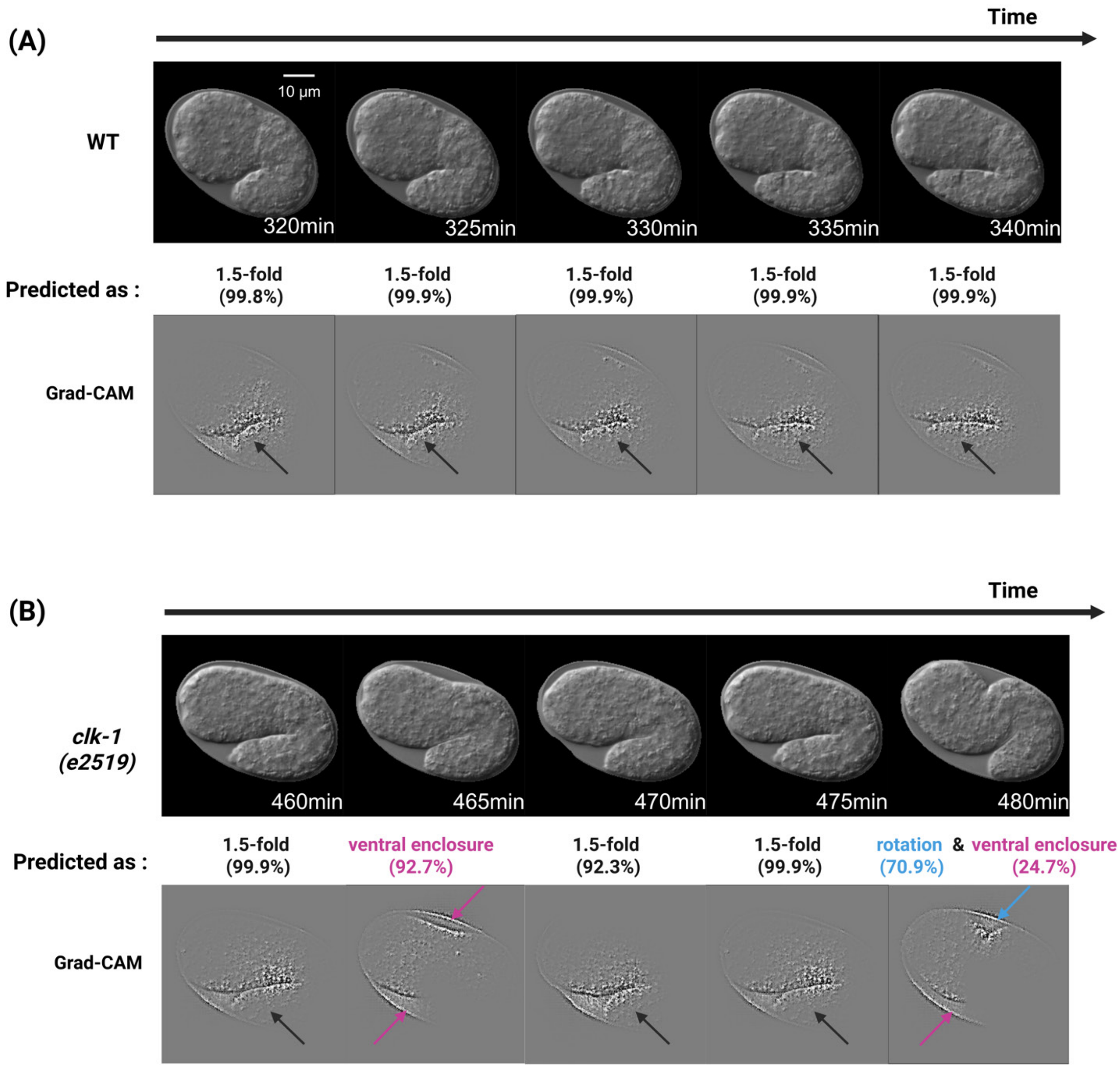


**Supplementary Figure S1.** *clk-1* embryos exhibit pronounced wriggling behavior at 1.5-fold stage. Arrows indicate Grad-CAM–highlighted regions. Pink and blue arrows mark areas of ventral enclosure and rotation, respectively. Scale bar: 10 μm. **(A)** Example of a WT embryo during the 1.5-fold stage. The top row shows the embryo image processed by ResU-Net, the middle row presents the predicted probabilities, and the bottom row displays the Grad-CAM visualization. **(B)** Example of a *clk-1* embryo during the 1.5-fold stage. The top, middle, and bottom rows correspond to the ResU-Net–processed image, predicted probabilities, and Grad-CAM results, respectively.

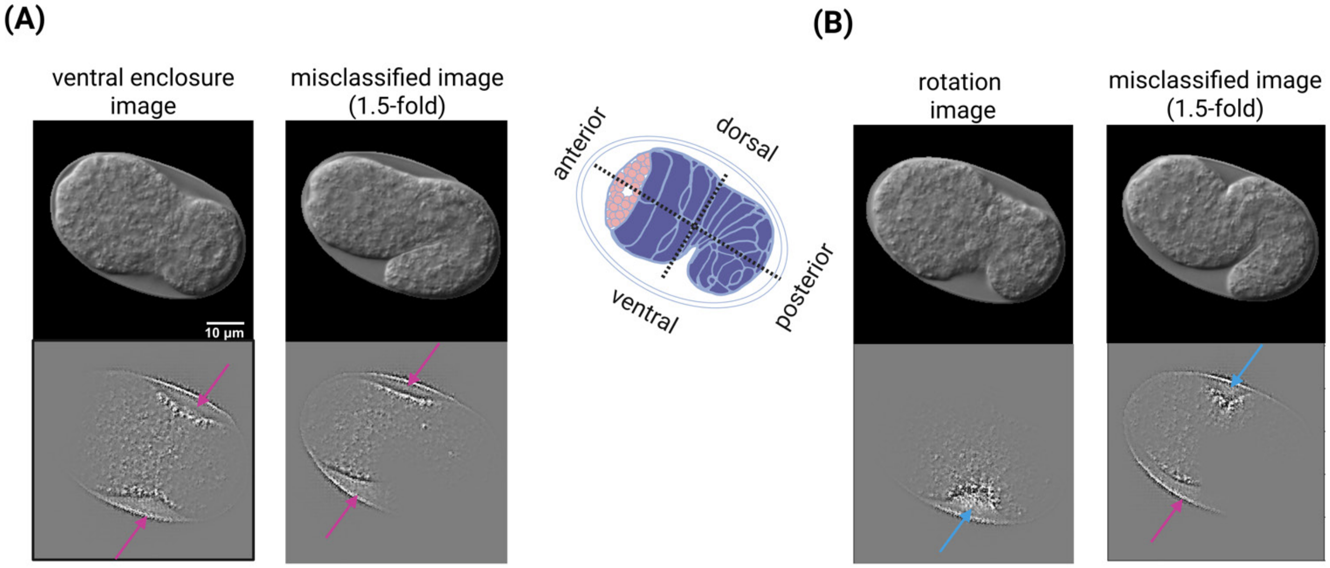


**Supplementary Figure S2.** exhibit pronounced wriggling can cause the AI to misinterpret the embryonic body axis. The middle panel illustrates the schematic representation of the embryonic axis. Scale bar: 10 μm. **(A)** Example of a misclassification in ventral enclosure: the left image shows a correctly predicted ventral enclosure stage, whereas the right image shows an incorrect prediction caused by axis misinterpretation. **(B)** Example of a misclassification in rotation: the left image shows a correctly predicted rotation stage, whereas the right image shows an incorrect prediction due to axis misjudgment.

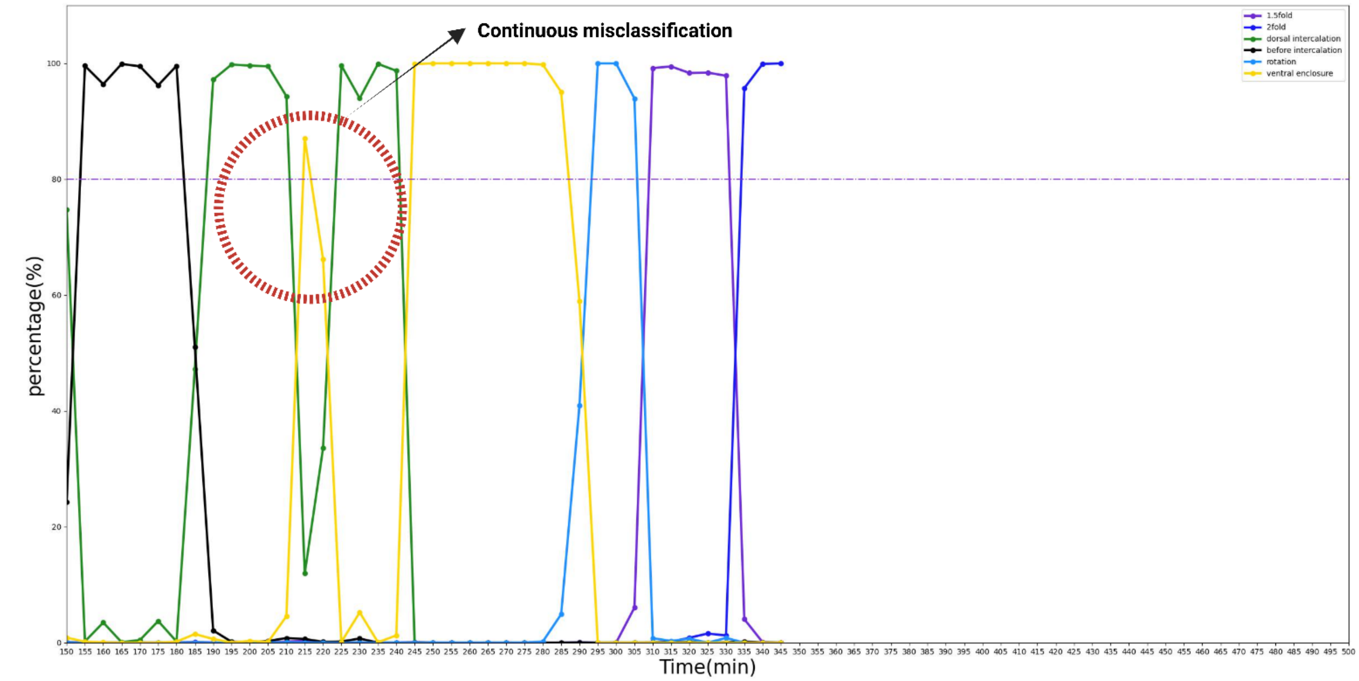


**Supplementary Figure S3.** Screened timeline examples that show continuous misclassification Different colors of black, green, yellow, sky blue, purple, and dark blue represent "before intercalation," "dorsal intercalation," "ventral enclosure," "rotation," "1.5-fold," and "2-fold" stages. The red circle indicates the location where a continuous misclassification occurred.

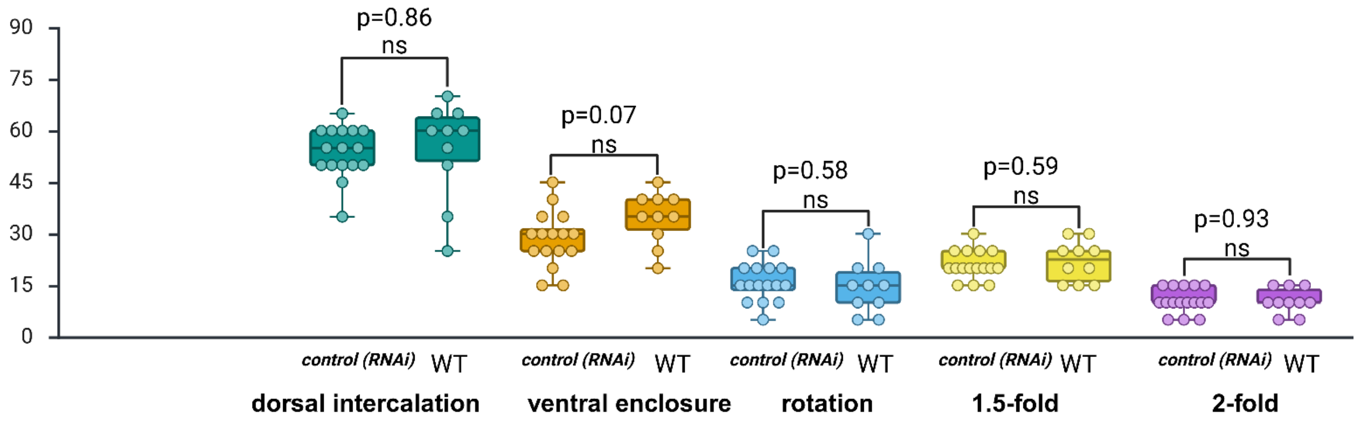


**Supplementary Figure S4.** Comparison of embryonic stage durations between *control(RNAi)* and WT groups. No significant differences were observed across the five developmental stages. Each dot represents the duration of a single embryo for the corresponding developmental stage.

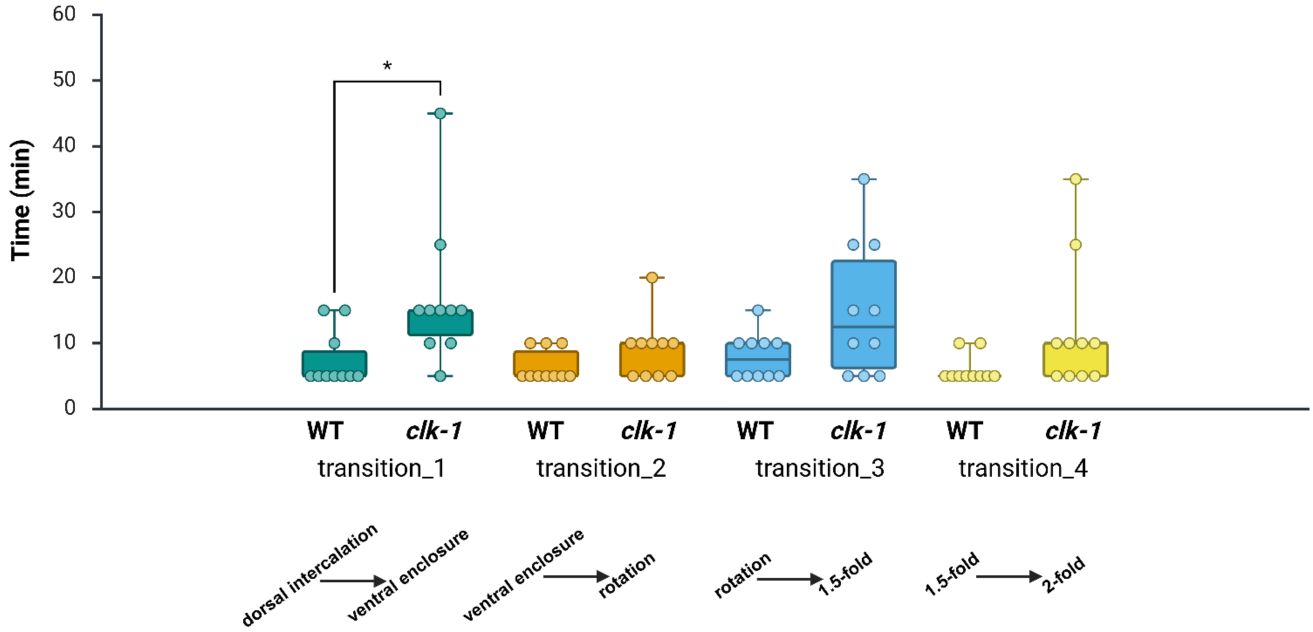


**Supplementary Figure S5.** Comparison of transition periods between *WT* and *clk-1* groups. The transition period from dorsal intercalation to ventral enclosure showed a marked delay * $p < 0.05$; Student's T-test. Each dot represents the duration of a single embryo for the corresponding developmental stage.

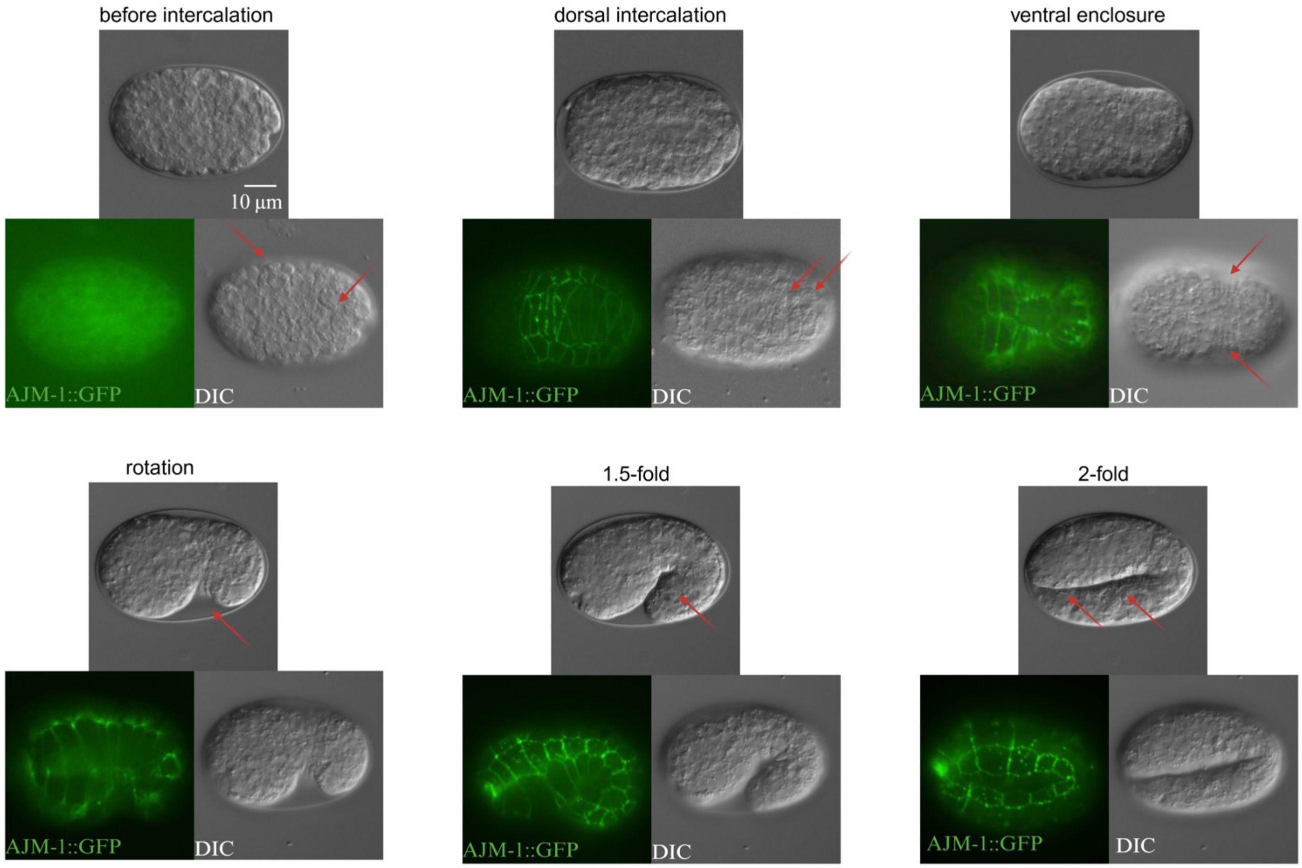


**Supplementary Figure S6** The *ajm-1::GFP* reporter strain was used to confirm the defining features of each embryonic stage before collecting training data. Scale bar: 10 μm. The images above represent the focus standard used for training, the bottom-left images show the embryos under a fluorescence microscope, and the bottom-right images illustrate the focus adjusted to the embryo surface. The red arrows indicate the representative features of each embryonic stage.

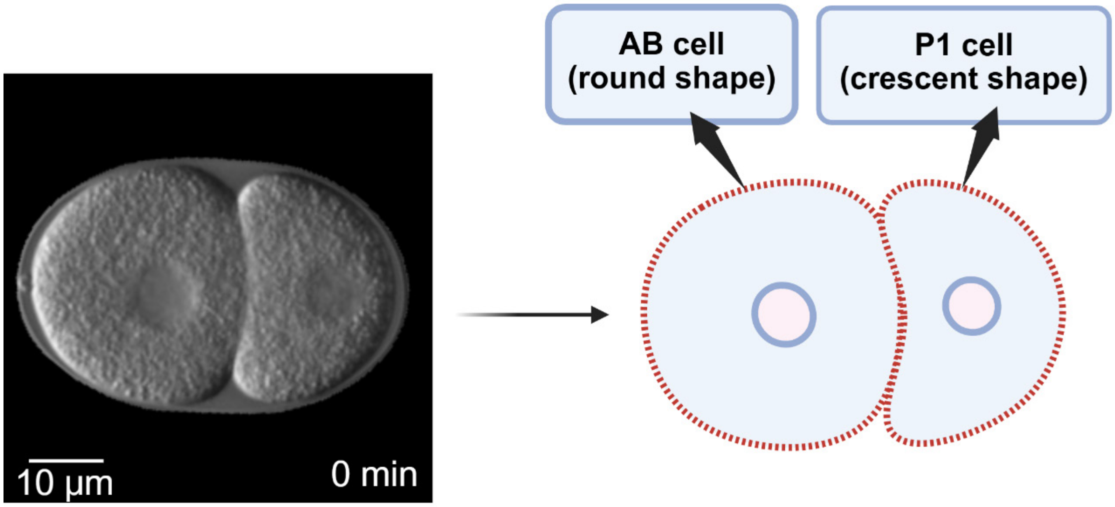


**Supplementary Figure S7.** Schematic diagram of the two-cell stage at the beginning of the experiment (0 min). Scale bar: 10 μm.

**Supplementary Figures S8.** Examples of calculating the duration of each embryonic stage in highly unstable predicted timelines

Example 1

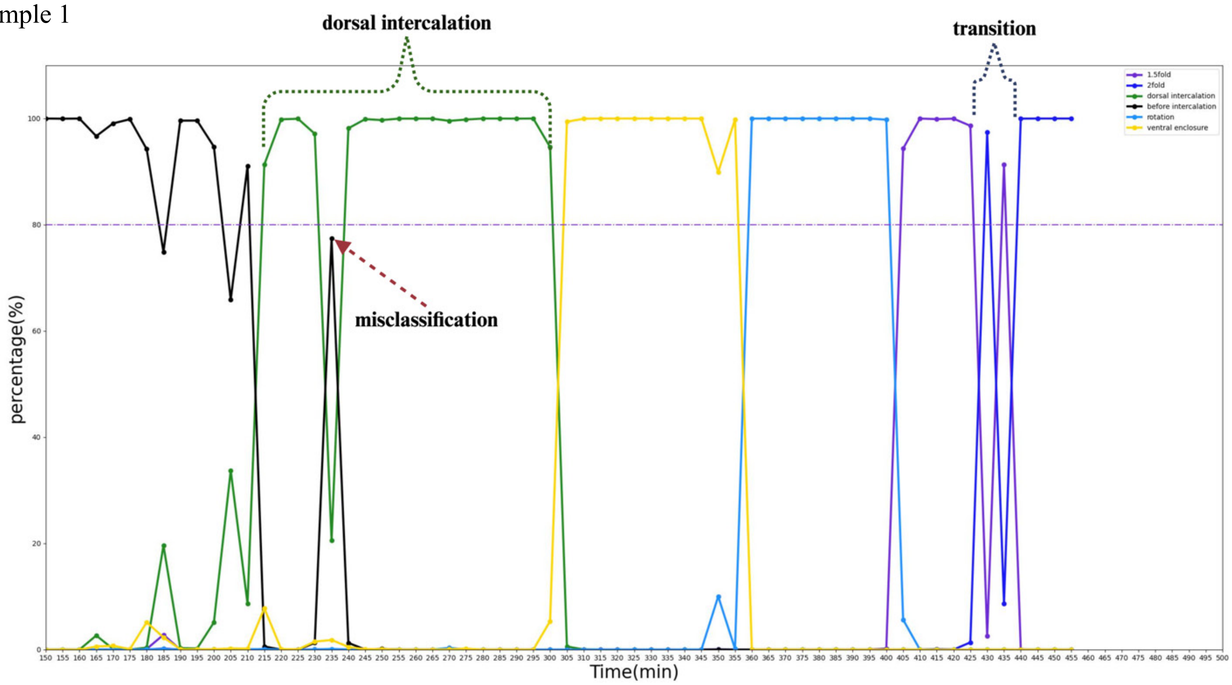


Example 2

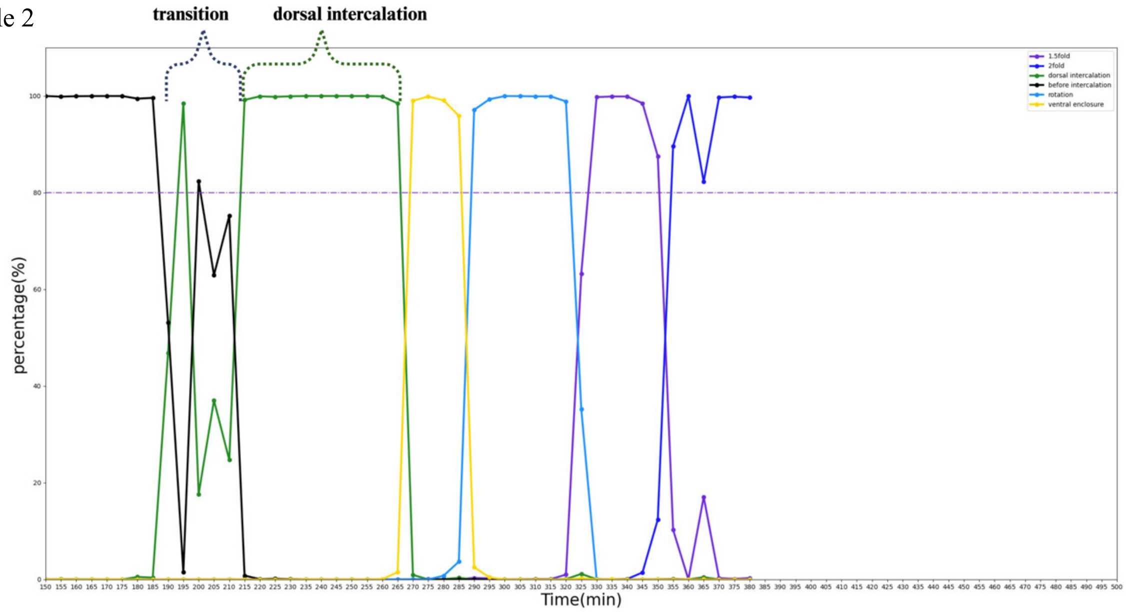

Example 3

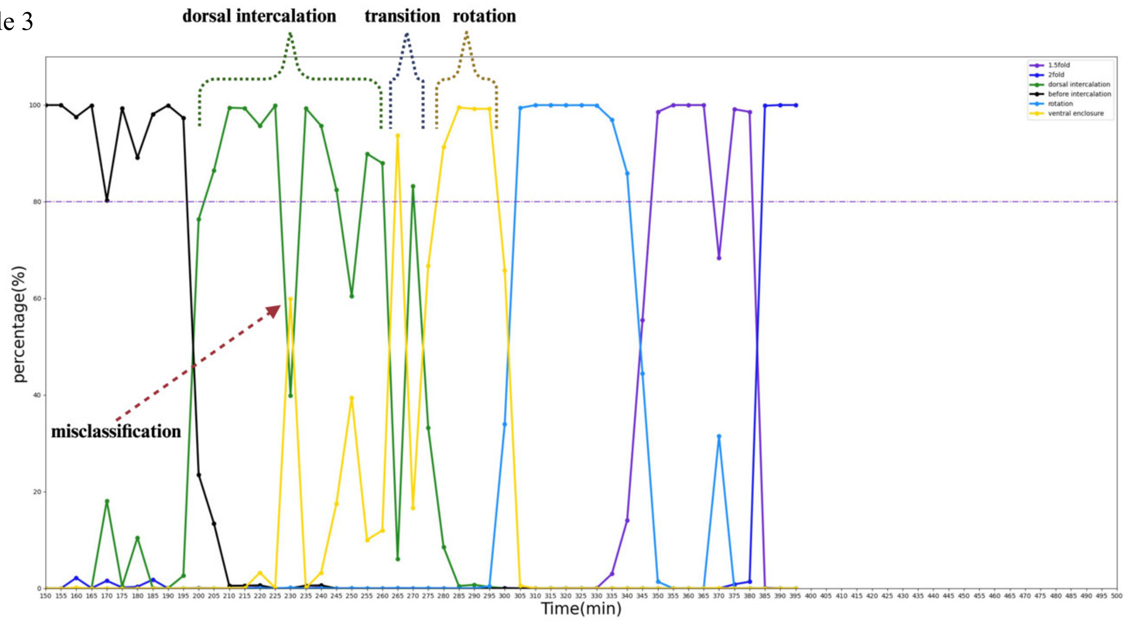


Example 4

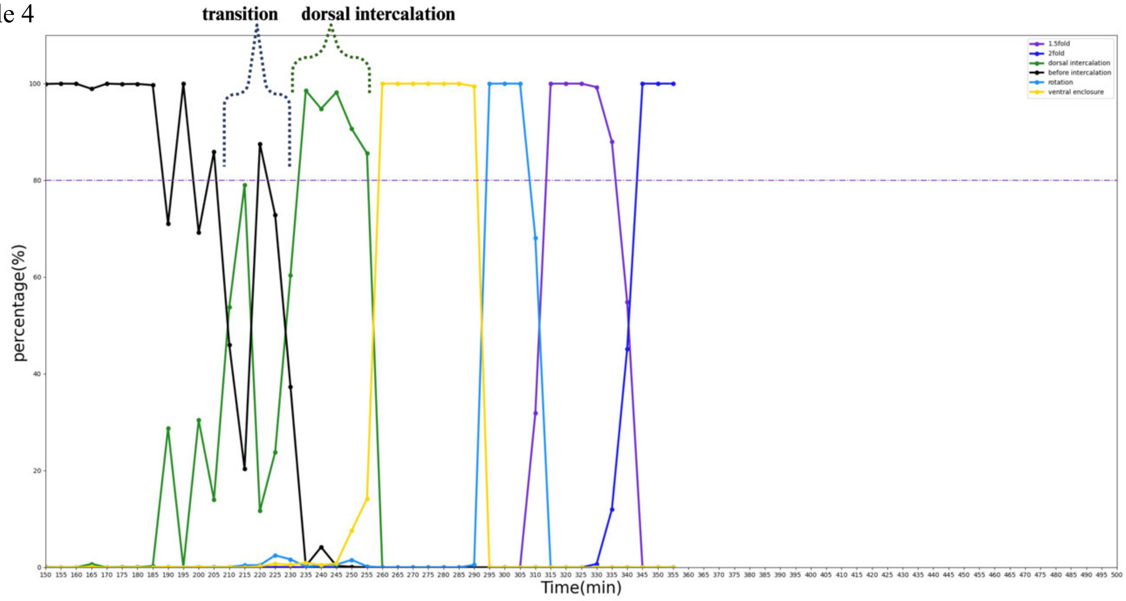

Example 5

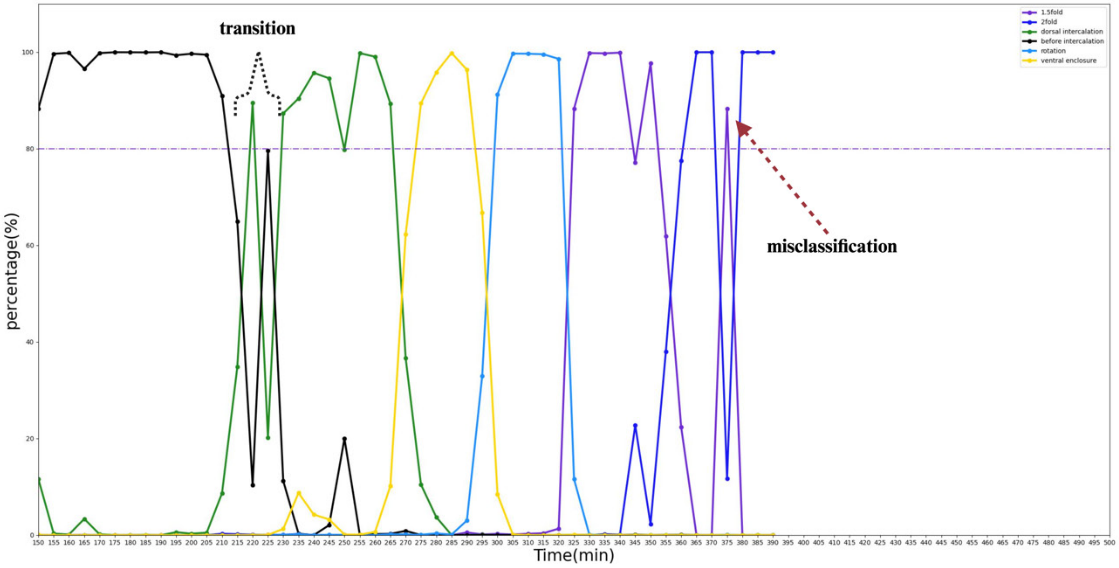
transition
misclassification
1.5fold
2fold
dorsal intercalation
before intercalation
rotation
ventral enclosure
percentage(%)
0
20
40
60
80
100
Time(min)
150 155 160 165 170 175 180 185 190 195 200 205 210 215 220 225 230 235 240 245 250 255 260 265 270 275 280 285 290 295 300 305 310 315 320 325 330 335 340 345 350 355 360 365 370 375 380 385 390 395 400 405 410 415 420 425 430 435 440 445 450 455 460 465 470 475 480 485 490 495 500
