# Supplementary material for: Temporal Analysis of Embryonic Epidermal Morphogenesis in Caenorhabditis elegans
Source: Int J Mol Sci. 2025 Nov 6;26(21):10802. doi: 10.3390/ijms262110802 (PMC12608371; doi:10.3390/ijms262110802)
Supplement: Supplementary file 1 [file ijms-26-10802-s001.zip › Supplementary Table.pdf]

Supplementary Information:

Temporal Analysis of Embryonic Epidermal Morphogenesis in *Caenorhabditis elegans*

Supplementary Table S1. ResNet five-fold cross-validation accuracy and loss metrics

Fold	Validation Accuracy (%)	Validation Loss
1-fold	94.2%	0.16
2-fold	93.3%	0.22
3-fold	94.8%	0.17
4-fold	95.2%	0.16
5-fold	94.2%	0.20
Mean ± SD	94.3% ± 0.4%	0.18 ±0.03

Supplementary Table S2. The average time required for each stage of epidermal development without manual correction.

Strain	Dorsal intercalation (min)	Ventral enclosure (min)	Rotation (min)	1.5-fold (min)	2-fold (min)
control(RNAi)	53.13 ± 1.46	28.43 ± 2.02	15.93 ± 1.38	20.93 ± 1.04	10.62 ± 0.89
leo-1(RNAi)	64.00 ± 4.18*	44.50 ± 4.80 **	28.50 ± 3.08 **	21.00 ± 2.56	11.00 ± 1.80
ajm-1(RNAi)	49.50 ± 4.00	25.50 ± 3.37	21.00 ± 3.23	27.50 ± 2.39 *	14.50 ± 2.23 *
tes-1(RNAi)	48.50 ± 6.89	28.50 ± 3.73	15.00 ± 2.47	21.00 ± 1.25	20.50 ± 1.74 ****
WT	53 ± 4.90	34.50 ± 2.41	14.5 ± 2.41	22.00 ± 1.86	10.50 ± 1.17
clk-1(e2519)	96.50 ± 6.41 ***	126.00 ± 24.45 **	76.00 ± 26.41 *	70.50 ± 8.11 ***	28.00 ± 3.43 ***

foot note: The bule color represents the time required of epidermal development stages that was calculated without manual correction.

* $p < 0.05$; ** $p < 0.01$; *** $p < 0.001$; **** $p < 0.0001$
